# Feasibility of orbital friction stir welding on clad pipes of API X65 steel and Inconel 625

**DOI:** 10.1038/s41598-023-37913-4

**Published:** 2023-07-01

**Authors:** C. V. Amavisca, L. Bergmann, C. R. de L. Lessa, J. G. Schroeder, F. D. Ramos, G. V. B. Lemos, A. Reguly, B. Klusemann

**Affiliations:** 1 Institute for Production Technology and Systems, Leuphana University Lüneburg, Leuphana University Lüneburg, Universitätsallee 1, 21335 Lüneburg, Germany; 2 Federal University of Rio Grande do Sul (UFRGS), Av. Paulo Gama, 110, Porto Alegre, 90040-060 Brazil; 3 Institute of Materials Mechanics, Solid State Materials Processing, Helmholtz-Zentrum Hereon, Max-Plank-Straße 1, 21502 Geesthacht, Germany; 4 Federal Institute of Rio Grande do Sul (IFRS), R. Avelino Antônio de Souza 1730, Caxias do Sul, 95043-700 Brazil; 5 Federal University of Santa Maria (UFSM), Rod. Taufik Germano, 3013, Cachoeira do Sul, 96503-205 Brazil

**Keywords:** Materials science, Structural materials, Metals and alloys

## Abstract

Orbital friction stir welding (FSW) has been applied to clad pipes, which is certainly of interest to the oil and gas industry. In this context, an FSW system capable of performing sound joints in one pass with full tool penetration was developed. Orbital FSW was executed in 6 mm thick API X65 PSL2 steel clad pipes with 3 mm thick Inconel 625 using a polycrystalline cubic boron nitride (pcBN) tool. The metallurgical and mechanical properties of the joints were investigated. Sound joints with axial forces of 45–50 kN, tool rotational speeds of 400–500 rpm, and a welding speed of 2 mm/s were obtained, illustrating that the developed system can perform FSW joints without volumetric defects.

## Introduction

The main challenges of the oil and gas industry are related to the exploration of deep and ultra-deep wells, which present an aggressive environment with salt and gasses like H_2_S and CO_2_. In this context, the usage of carbon steel pipes clad in a corrosion resistant alloy (CRA) has proven to be a suitable option to meet design requirements at low manufacturing costs^1–3^. However, pipeline welding currently used by the oil and gas industry, such as fusion-based welding, often involves high temperatures and can cause metallurgical issues owing to dissimilar joints (e.g., clad pipes), thus resulting in solidification cracking, hard microstructures at the interface, high tensile residual stresses and excessive carbon diffusion that may compromise the joint performance^4,5^.

Orbital friction stir welding (FSW), a solid-state joining process, is recognized as a suitable alternative that has the potential to minimize the challenges typically found in fusion-based welding since it is performed at lower temperature and shorter processing time, which results in reduced energy input, avoiding (or minimizing) most of the above-mentioned issues^6,7^. FSW uses the friction energy produced between the materials to be joined and a non-consumable rotating tool. Two approaches are used in orbital FSW: in the first, the pipe rotates while the rotating tool remains stationary; and in the second, the entire tool head rotates while the pipe remains stationary^8^. As for the classical FSW, severe plastic deformation and material flow occur along the welding path. In short, the material is transported from the front of the tool to the trailing edge, later forged, producing the joint^9–11^.

Although FSW is successfully applied to sheets of various materials, orbital FSW involves challenges to be overcome since there are difficulties associated with the clamping device^8,12^. Challenges of FSW to complex and circular-shaped joints were recently reviewed by Senthil et al.^8^, where the clamping system was one of the most significant factors for the welding process itself. For instance, due to the high forces during FSW, internal support of the pipes is required. Another difference when welding pipes is the contact of the tool with the parts to be joined, e.g., depending on the diameter of the pipe, the curvature of the pipe results in a non-complete contact of the tool shoulder with the workpiece, thus influencing the material flow and heat generation^12,13^. Senthil et al.^8^ also pointed out that before FSW can be considered for industrial pipe joining, a deep understanding of the influence of the clamping system is essential.

Overall, only a few studies were performed on orbital FSW on pipeline steels using different approaches to achieve sound joints. Feng et al.^14^ studied orbital FSW welding of an API X65 steel, where sound welds were obtained using a specially designed portable FSW system. The joints showed slightly higher mechanical strength and superior impact resistance than the base material (BM). Giorjao et al.^15^ performed orbital FSW in an 8 mm thick super duplex stainless steel pipe in force control mode and total tool penetration. Hardness and tensile tests showed a hardness increase in the stir zone (SZ) and failure on the BM. However, orbital FSW in clad pipes is scarce, but surely needed for industrial application.

In this work, orbital FSW on clad pipes of API X65 steel and alloy 625 was performed. A very rigid clamping system to handle the forces and torque during the process was employed to enable orbital FSW, ensuring a constant weld quality for the pipes. Thus, one pass orbital FSW process with full tool penetration was successfully performed, and the resulting metallurgical and mechanical properties are discussed in the following.

## Materials and methods

Orbital FSW was performed on API 5L X65 PSL2 high-strength low-alloy (HSLA) steel pipes with an inner clad layer (3 mm) of Inconel 625. Both pipe materials are metallurgically bonded. In other words, the bond between the materials is overall characterized by an interdiffusion. The base material (BM) was provided by Butting GmbH & Co., and the cladding process used on the pipes were carried out by hot-roll bonding^16,17^. The chemical composition of the API X65 PSL2 steel and Inconel 625 was determined by optical emission spectrometry (OES) and energy dispersive spectroscopy (EDS), respectively, and these results are given in Table 1.

**Table 1:** Chemical composition of the clad pipe (wt%).

Element	Ni	Cr	Mo	Fe	Si	Mn	C	Al	P	S
API X65 PSL2	0.15	0.16	–	Bal	0.32	1.58	0.05	0.04	0.01	0.002
Inconel 625	58.19	22.09	7.51	4.46	–	0.37	–	1.18	–	–

The pipes had a total wall thickness of 9 mm and an external diameter of 310.5 mm. Single pass orbital FSW was performed on butt sections of the pipes with the pcBN tool of MegaStir™, designated as Q70, i.e., 70 wt% cBN and 30 wt% W–Re as the binder. The tool shows a convex radius shoulder with a diameter of 25 mm and a stepped spiral probe with a length of 8.5 mm.

**Figure 1:**
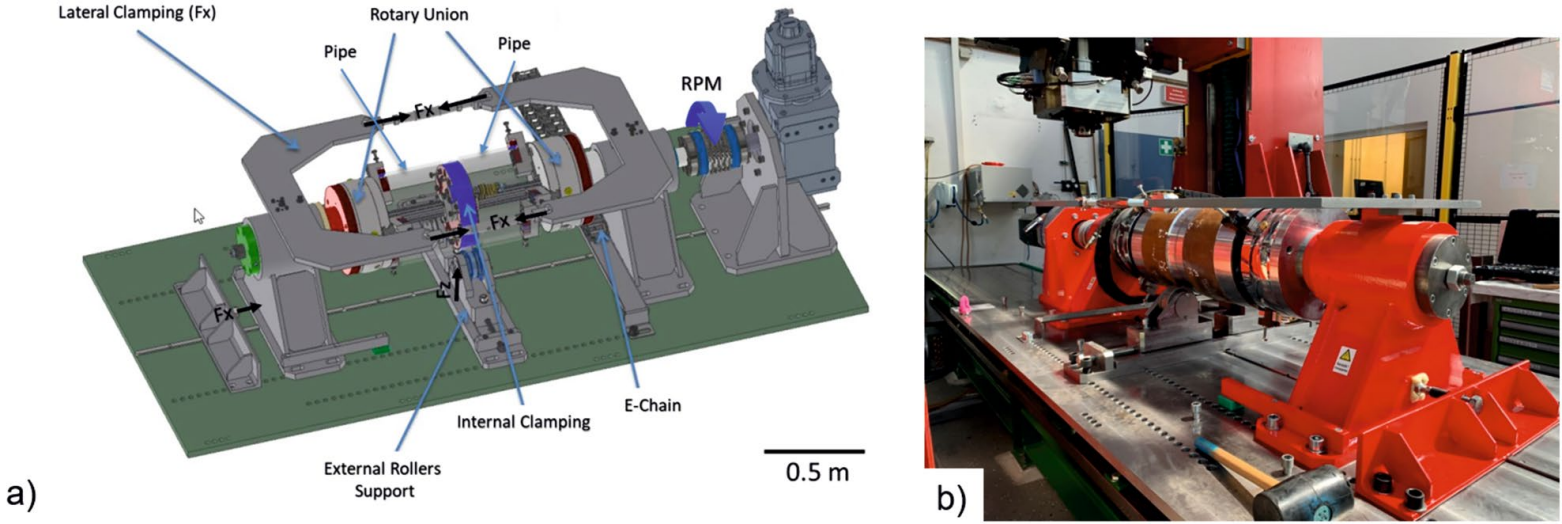
(**a**) Schematic drawing and (**b**) image of the clamping system developed for orbital friction stir welding.

A manipulator that is rigid enough to absorb the forces of the FSW process and apply the required torque to the pipes was developed to ensure a constant welding speed and guarantee proper clamping. A schematic drawing of the employed clamping system is shown in Fig. 1a. An internal clamping is required to prevent local collapse of the pipe during welding and guarantee the centering of the pipes and in this regard the quality of the weld. The external rollers support the force (Fz) applied by the FSW tool and avoid pipe deflection. The lateral clamping ensures full contact of the parallel cross-section of the two pipes to be welded. The rotatory union rotates the pipes at controlled rotational speed, determining the resultant welding speed, where the rotating FSW tool is kept at a fix spatial position. Figure 1b shows two sections of pipe set in the clamping system ready to be welded.

The main features of the Hereon portal FSW system are rotational speed up to 6000 rpm, maximum axial force of 60 kN and maximum torque of 140 Nm. Argon shielding gas was used to protect the top surface of the weld around the pcBN tool from oxidation. During welding, the tool stays stationary, and the pipe rotates. When the pipe starts to rotate, the plasticized material is stirred around the rotating stationary tool producing the welded joint, i.e., the pipe movement is equivalent to the tool traverse speed in FSW of sheets.

An inherent feature of FSW is an exit hole left at the end of the process. Methods to avoid the exit hole, which could be considered as a substantial defect in pipes, are presented in the literature, including devices to eliminate^14,18^ or to fill it^19^. However, this aspect goes beyond the focus of this study and will therefore not be considered further. To demonstrate the capability of the employed clamping system in producing sound joints in clad pipes, two different process parameters were investigated in this study, selected based on previous work on X65 steel sheets clad by welding overlay with Inconel 625^4^. In this previous work, the force was varied between 60 and 40 kN, leading to good joints. Therefore, in this work, the intermediate value was chosen as the initial value (50 kN) and subsequently adopted to decrease by 10% to evaluate the corresponding response, see Table 2.

**Table 2:** Investigated FSW process parameters.

Weld	Force (kN)	Rotational speed (RPM)	Welding speed (mm/s)	Energy input (kJ/mm)
I	50	500	2	2.36
II	45	400	2	2.05

Microstructural analysis of the weld zones was carried out using Keyence VHX-6000 optical microscopy (OM) and an FEI Quant 650 FEG scanning electron microscopy (SEM) equipped with EDAX Apollo X EDS system and EDAX velocity EBSD camera. EDS was performed to analyze the chemical composition in the BM, starting on the steel side, passing through the interface zone, and ending on the Inconel side. In addition, EDS was used to evaluate the presence of M (C, N) carbonitrides and intermetallic compounds. Samples were prepared using standard metallography, followed by two-step etching. To reveal the microstructure of the X65 steel and alloy 625, a 2%-vol Nital and an Adler’s solution (25 ml H_2_O, 3 g CuCl_2_, 15 g FeCl_3_, 50 ml HCl) were used, respectively. For the electron backscatter diffraction (EBSD) analysis, after the standard metallography, samples were prepared with a frequency of 50 Hz for 1 h in the VibroMet 2 Vibratory Polisher machine. The EBSD investigation was performed using a step size of 0.25 μm, and the acquired data were analyzed using the TSL OIM 7.3 software. Struers DuraScan 70 was used to perform Vickers microhardness measurements in the cross-section using a load of 500 g (HV 0.5) with distances between indentations of 0.5 mm. The temperature was measured during FSW by a thermocouple (Type K) inserted in the tool shoulder through the locking collar. The thermocouple was held against the pcBN tool, allowing consistent temperature readings.

## Results and discussion

### Welding process

**Figure 2:**
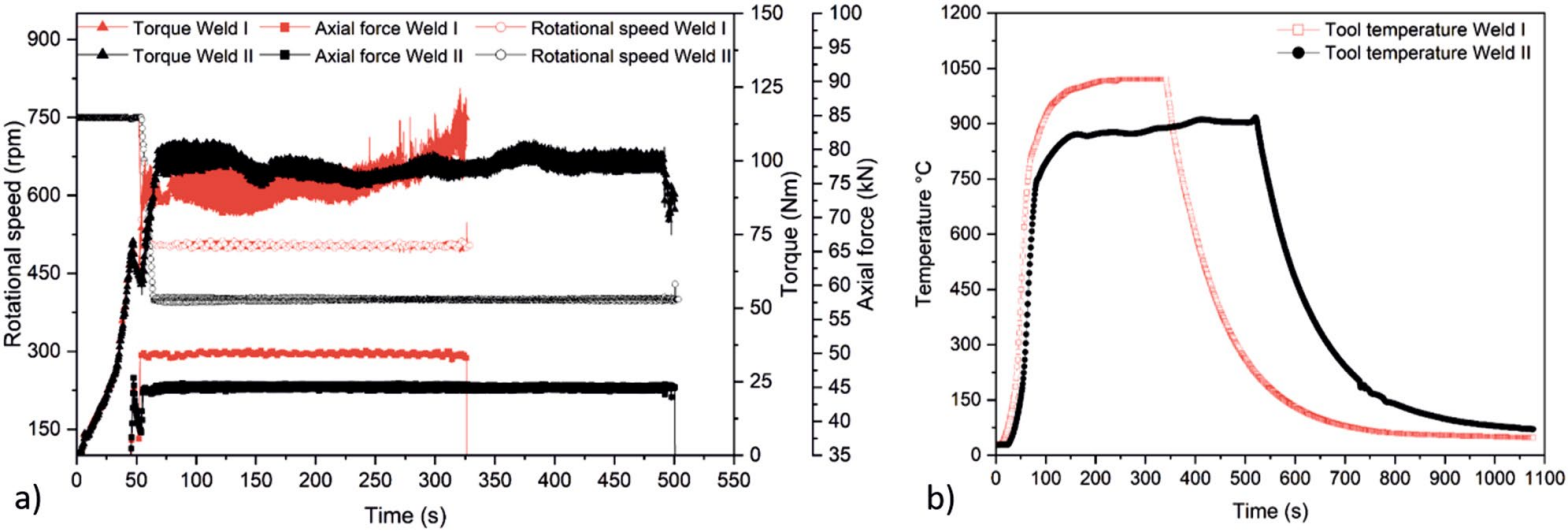
(**a**) Rotational speed, axial force, and torque during the orbital FSW process for both pipes. (**b**) Tool temperature measured within the tool during the processes.

In the initial welding phase, i.e., during the initial plunging stage, the rotational speed was set to 750 rpm (Fig. 2a). As soon as the material around the probe is plasticized, the rotational speed is set to the target values, i.e., to 500 rpm and 400 rpm for Welds I and II, respectively, see Table 2. At this point, the pipe rotates at a welding speed of 2 mm/s. The process is force-controlled, with forces of 50 kN and 45 kN for Weld I and II, respectively. Since the process parameters in Weld II led to lower temperatures, Fig. 2b shows the higher material resistance from the machine response in terms of a slightly higher torque compared to Weld I. It is important to notice that the welds have different lengths, as Weld II had to be stopped prematurely after 327 s due an overheating in the machine. However, the welding length is more than sufficient for an in-depth analysis of the resulting properties.

### Microstructural characterization

**Figure 3:**
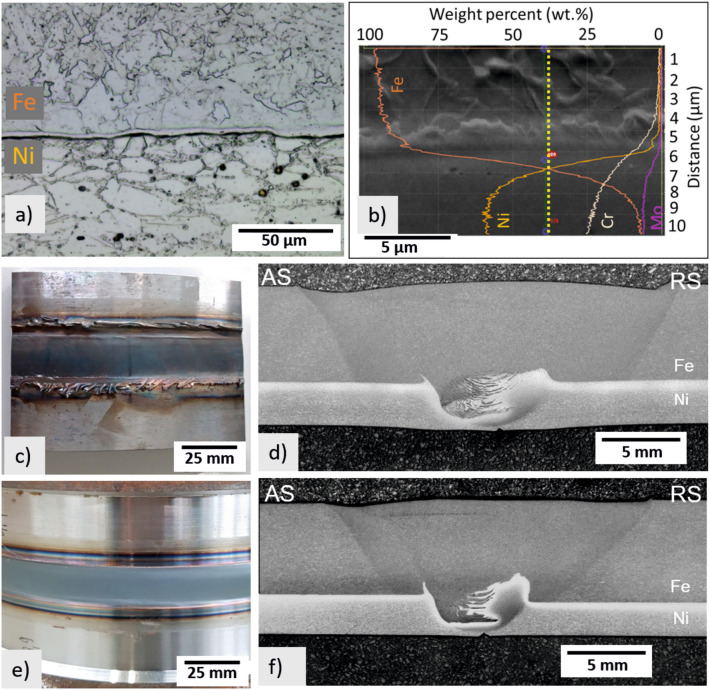
Microstructural analysis of the clad pipe BM, above the API X65 steel and below the interface of the Inconel 625; (**b**) EDS line scan utilized to identify the chemical composition along the steel/Inconel interface in the BM; (**c**,**d**) top surface appearance and macrostructure of Weld I and (**e**,**f**) top surface appearance and macrostructure of Weld II.

The microstructural features of the BM are shown in Fig. 3. The X65 steel is composed of acicular ferrite (AF), quasi-polygonal ferrite (QPF), polygonal ferrite (PF), carbides, and martensite/austenite (M/A) phase, while the CRA layer (Inconel 625) presents a microstructure of elongated austenitic grains and M(C, N) carbonitrides. In addition, carbides along grain boundaries can be observed, which are typically identified as M_6_C and M_23_C_6_ (Fig. 3a). Due to the hot roll-bonding process used to produce the clad pipes involving high deformation at elevated temperature, substitutional inter-diffusion of Ni and Cr to the steel side and Fe to the CRA side occurred, besides interstitial diffusion of C from steel to the CRA. That results in austenitic steel in the interface due to the stabilizing effect of Ni^16,20^. Figure 3b shows the EDS line scan along the interface of the pipe, presenting a clear transition from steel (Fe) to Inconel (Ni and Cr). Microhardness tests of the BM led to 210 HV, 249 HV, and 318 HV for steel, interface, and Inconel 625, respectively.

An overview of the two welded joints is also provided in Fig. 3. Weld I, Fig. 3c, resulted in a surface with more flash and a larger heat-affected zone (HAZ), Fig. 3d, when compared to that of the Weld II (Fig. 3e,f). Latter is expected due to the higher energy input, see Table 2, and the resulting higher temperature (Fig. 2b). The temperatures in the tool reached a maximum of 1020 °C for Weld I and 916.5 °C for Weld II.

**Figure 4:**
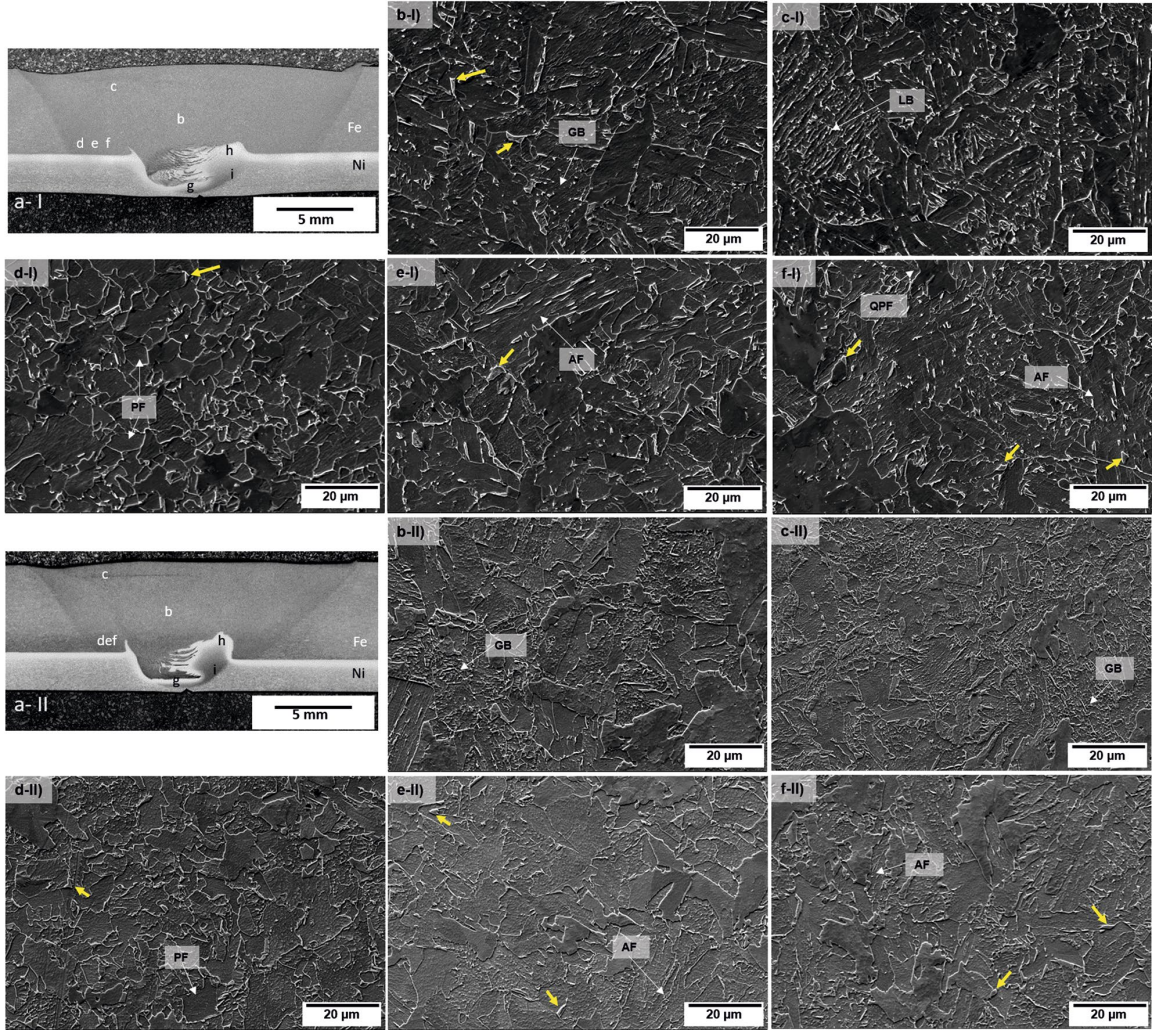
(**a**) Macrographs of different zones in the X65 steel side for Weld I and Weld II. (**b**) SZ_X65_, (**c**) HZ_X65_, (**d**) OHAZ_X65_, (**e**) MHAZ_X65_ and (**f**) IHAZ_X65_. The yellow arrows point out the M/A phase. In the micrographs, the different zones analyzed on the Inconel side (**g**,**h**,**i**) are indicated already, see Fig. 5.

The joint microstructure can be divided into three core regions: steel side, Ni-based alloy 625 side, and interface (Fig. 4a). On the steel side, the SZ_X65_ and hard zone (HZ_X65_) are observed. Similarly to other studies^21–23^, the HAZ_X65_ can be subdivided into three sub-regions with different microstructures: the outer HAZ (OHAZ_X65_), middle HAZ (MHAZ_X65_), and the inner HAZ (IHAZ_X65_). As observed in Fig. 2b, the X65 steel experienced a peak temperature above Ac3 (i.e., the critical temperature in the Fe–C diagram) during welding; the microstructure is transformed into the austenite, and according to the cooling rate and the peak temperature, the austenite is transformed to different microstructures in the form of martensite, bainite, ferrite, carbide or a combination of them^22^.

**Figure 5:**
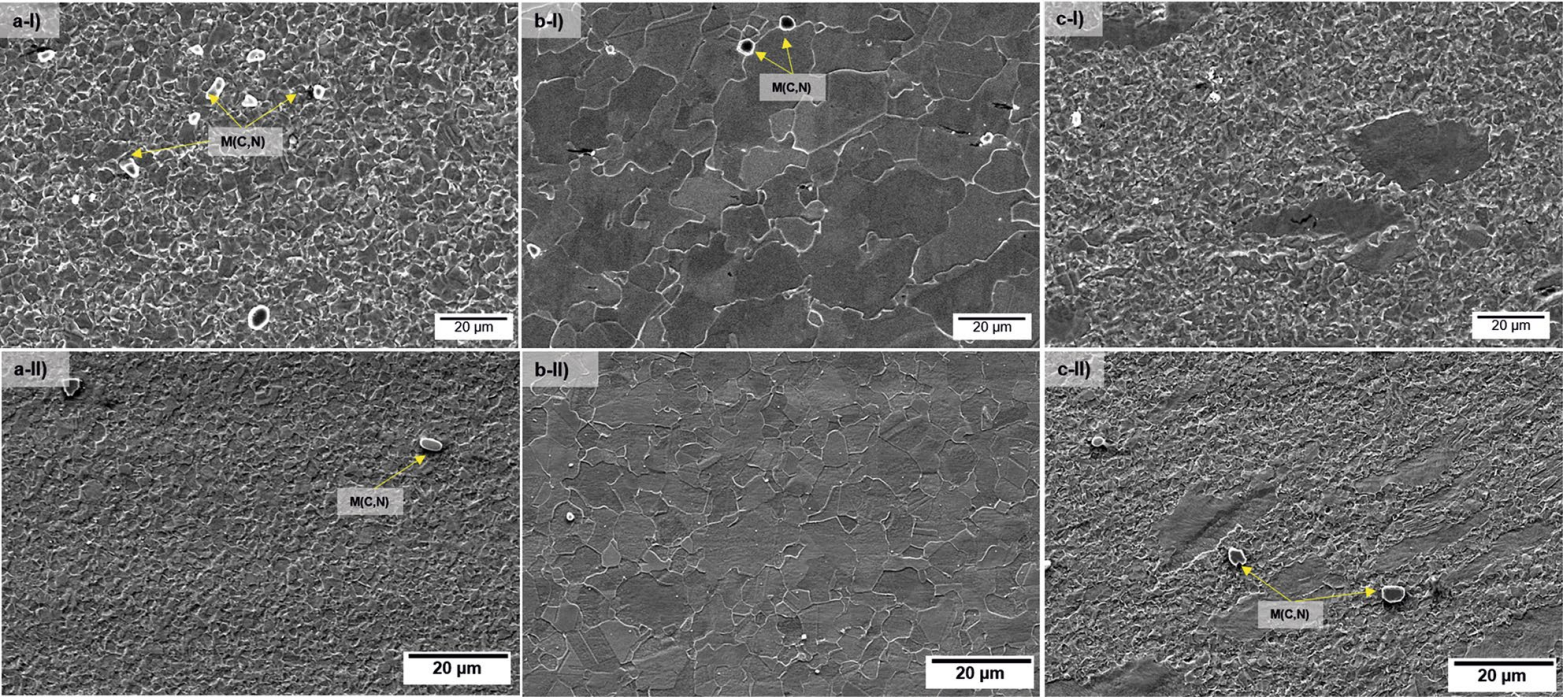
Optical micrographs of different zones in Inconel 625 for Weld I and Weld II: (**a**) SZ1_625_ showing refined austenitic grains, (**b**) SZ2_625_ shows characteristic coarser austenitic grains and (**c**) TMAZ_625_ deformed grains. SZ1_625_, SZ2_625_, and TMAZ_625_ are indicated in the macrographs of both welds as (**g**–**i**) in Fig. 5.

The SZ_X65_, Fig. 4b, comprises AF and granular bainite. Additionally, since HZ_X65_ may have experienced the highest deformation, peak temperatures, and cooling rates during FSW^24^, a lath bainite microstructure (LB) is observed (Fig. 4c). The OHAZ_X65_, Fig. 4d shows a more refined equiaxed PF compared to the BM_X65_. In the MHAZ_X65_, the resulting microstructure is of QPF, PF, and AF (Fig. 4e). The IHAZ_X65_, Fig. 4f exhibits a mixed ferrite and bainite microstructure with finer prior austenite grain size and more PF than found in the SZ_X65_. M/A phase pointed out in Fig. 5, was also found in both welds. With the aid of EBDS, it was possible to quantify the percentage of face-centered-cubic structure in the steel, and according to the measurements, the BM showed 0.2%, Weld I 0.5%, and Weld II 0.2% of M/A.

Figure 5 presents the microstructural zones on the Inconel 625. Two sub-regions can be identified in the SZ_625_, i.e., SZ1_625_ and SZ2_625_. The FSW process led to significant grain refinement in the bottom of SZ1_625_ (Fig. 5a). In SZ2_625_, coarser and equiaxial austenitic grains are found (Fig. 5b), which suggests that the material was exposed to higher thermal cycles in relation to SZ1_625_, causing grain growth. The TMAZ_625_ presents a deformed microstructure following the probe flow pattern (Fig. 5c). The cabonitrides found in the welds are the M (C, N), inherent of the BM, with a high amount of niobium, molybdenum, or titanium. Precipitate transformation was not found in the welds in an SEM investigation. According to the literature, carbide precipitation is unlikely to occur during FSW process, as prolonged exposure at elevated temperatures seem to be required^5,20,25^.

**Figure 6:**
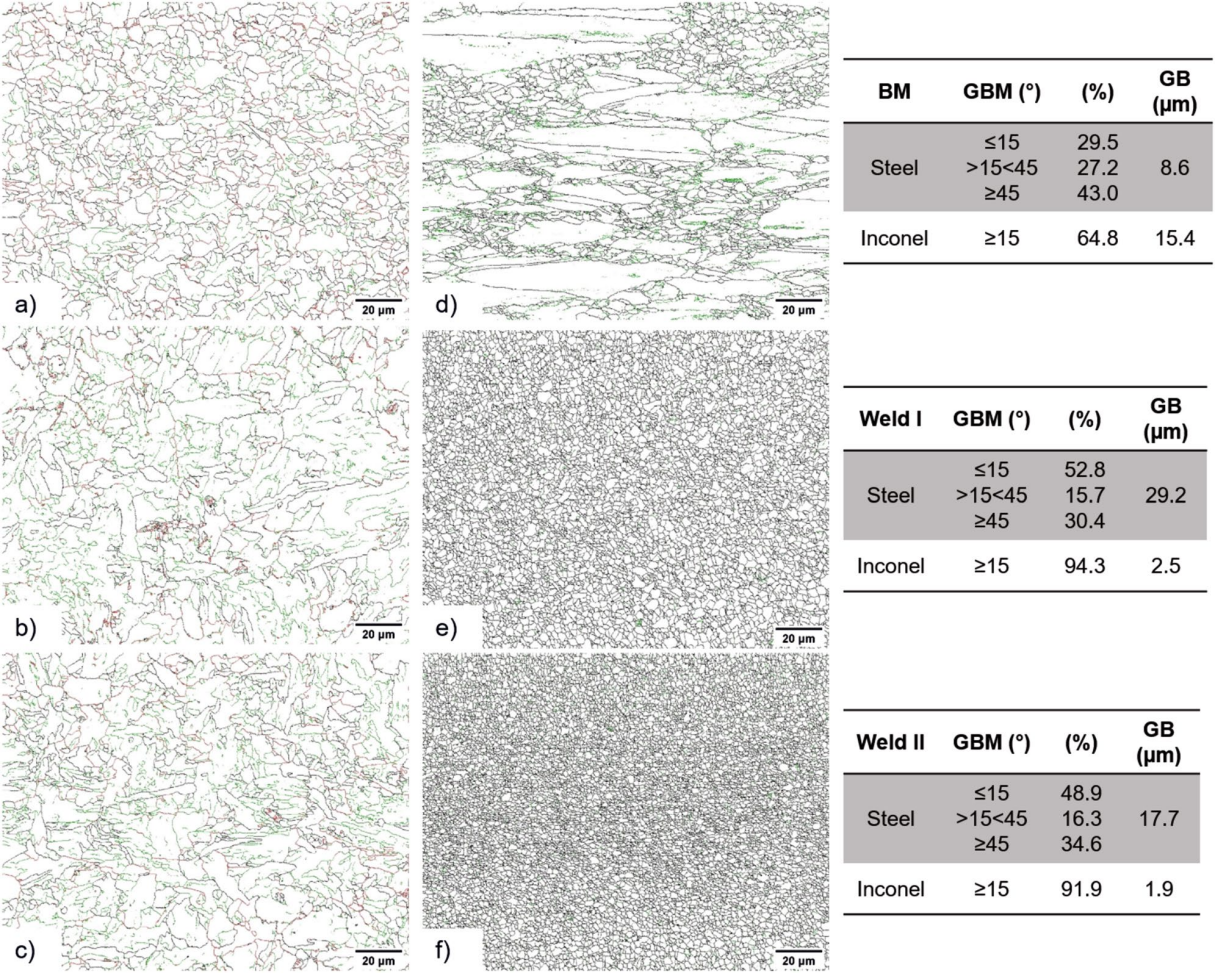
Grain boundary misorientation analysis via EBSD: (**a**–**c**) steel side and (**d**,**e**) Inconel 625 side; (**a**,**d**) BM; (**b**) SZ_X65_ and (**e**) SZ_625_ of Weld I and (**c**) SZ_X65_ and (**f**) SZ_625_ of Weld II.

Figure 6a–c shows the grain boundary misorientation (GBM) and average grain boundary  area (GB) distribution maps of the BM and SZs for Welds I and II for the X65 steel, in which the green lines represent very high-angle grain boundaries, i.e. grain boundary misorientation ≥ 45°, the red lines represent grain boundary misorientation between 15° and 45° and the black lines represent low-angle grain boundaries (LAGBs), i.e. < 15°. The steel SZ_X65_ showed around 51% of LAGBs and the BM reached 30% of LAGBs, indicating that a high number of dislocations were created in the SZ_X65_. The high amount of LABs found within the SZ_X65_ (~ 51%) and small peaks above 45° (~ 32%) suggests that the microstructure here has less acicular ferrite but more bainite^22,26^. The average GB area increased with the energy input, i.e. from 8.6 µm^2^ in the BM to 17.2 µm^2^ in Weld II and 29.2 µm^2^ in Weld I, see Fig. 6a–c.

It is important to point out that the recrystallization mechanism for austenite and ferrite phases occurs differently because of their distinct stacking fault energy (SFE)^27^. Ferrite has a high SFE and immediately underwent the recovery process; therefore, sub-grains were formed during hot deformation. Hence, a great number of LABs were generated within the grains. However, phases with low SFE, such as the austenite, can easily form recrystallization nuclei^28–30^. In Fig. 6d–f, the black line represents the HAGBs (≥ 15°), and the green line LAGBs (< 15°) for Inconel 625. The maps show that the concentration of HAGBs increases from the BM (64.8%) to the SZ_625_ (around 93.0%), indicating a fully recrystallized state. In addition, due to the inherent characteristics of FSW, such as severe plastic deformation, high strain rates, enough heat input, and fast cooling rate, thus an effective grain refinement is observed, from an average GB area of 15.4 µm^2^ in the BM to 2.5 µm^2^ and 1.9 µm^2^ in the SZ1_625_ of Weld I and Weld II, respectively.

**Figure 7:**
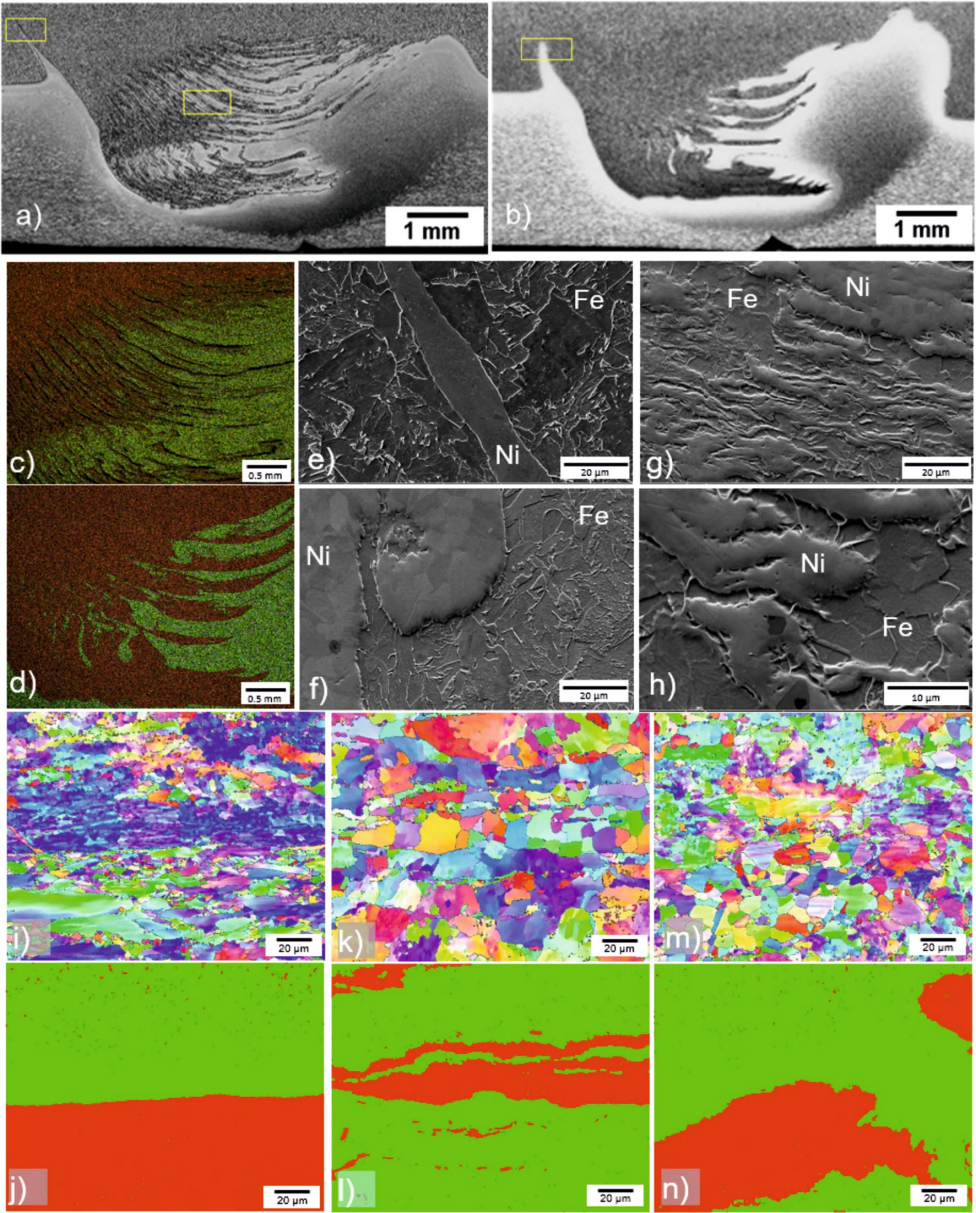
Different features in the interface of the clad pipes welded by FSW: SZ of Weld I (**a**) and of Weld II (**b**); EDS chemical composition of Weld I SZ (**c**) and Weld II (**d**), where green indicates Fe and red Ni; hook on AS of Weld I (**e**) and Weld II (**f**); detail of the alternated bands of Fe–Ni in the SZ in Weld I (**g**,**h**); EBSD analysis of interface in BM (**i**), Weld I (**k**) and Weld II (**m**); Identification of bcc (green) and fcc (red) structures of corresponding EBSD micrographs is shown for the interface of BM (**j**), Weld I (**l**) and Weld II (**n**).

The interface of the welds is detailed in Fig. 7. At the joining temperature during FSW, as Inconel 625 and API X65 steel have the same crystal structure (fcc), similar melting points, and comparable flow stresses^31^, these characteristics allow the alloy 625 to flow around the probe and to drain into the steel, forming alternating bands of materials in the SZ, which is consistent with findings from Rodriguez & Ramirez^2^. This is also in agreement with other studies on dissimilar FSW welds^32^. For both welds, as can be seen in Fig. 7a,b,e,f, Inconel 625 hooks were formed in API X65 steel. The height of the hooks is around 1.38 mm (0.86 mm) on the AS and 0.84 mm (1.71 mm) on the RS for Weld I (Weld II). The shape of the alternating bands, might be related to the process parameters and heat input achieved. A higher heat input seems to contribute to a more homogeneous mixture of the materials. Figure 7c,d show EDS images, indicating Fe, and Ni rich regions. In Fig. 7g,h detailed images of the SZ are shown.

Results of EBSD analysis at the interface of the BM are shown in Fig. 7i and for Weld I, and Weld II in Fig. 7k,m, respectively. The corresponding separation into fcc (austenite) and bcc (ferrite) structures is presented in Fig. 7j,l,n. In the interface, the BM steel has a GB area of 10.9 µm^2^, and the clad bonding line is clearly observed. Weld I and Weld II present grain growth with an average  GB area of 18.7 µm^2^ and 14.18 µm^2^, respectively. It is interesting to notice that the grain growth in the X65 steel in the mixed interface is smaller than in the SZ_X65_ (29.2 µm^2^ and 17.7 µm^2^, for the welds I and II, respectively). After the FSW process, the line of the clad bonding cannot be easily distinguished anymore. The Inconel 625 grains in the mixed interface region underwent recrystallization and Weld I, with higher energy input showed an average grain size of 12.1 µm^2^ and Weld II, with lower energy input, an average grain size of 8.88 µm^2^. In both cases, the average grain size observed in the mixed interface presented a significant grain growth compared to the SZ1_625_ (~ 2.0 µm^2^).

### Microhardness

**Figure 8:**
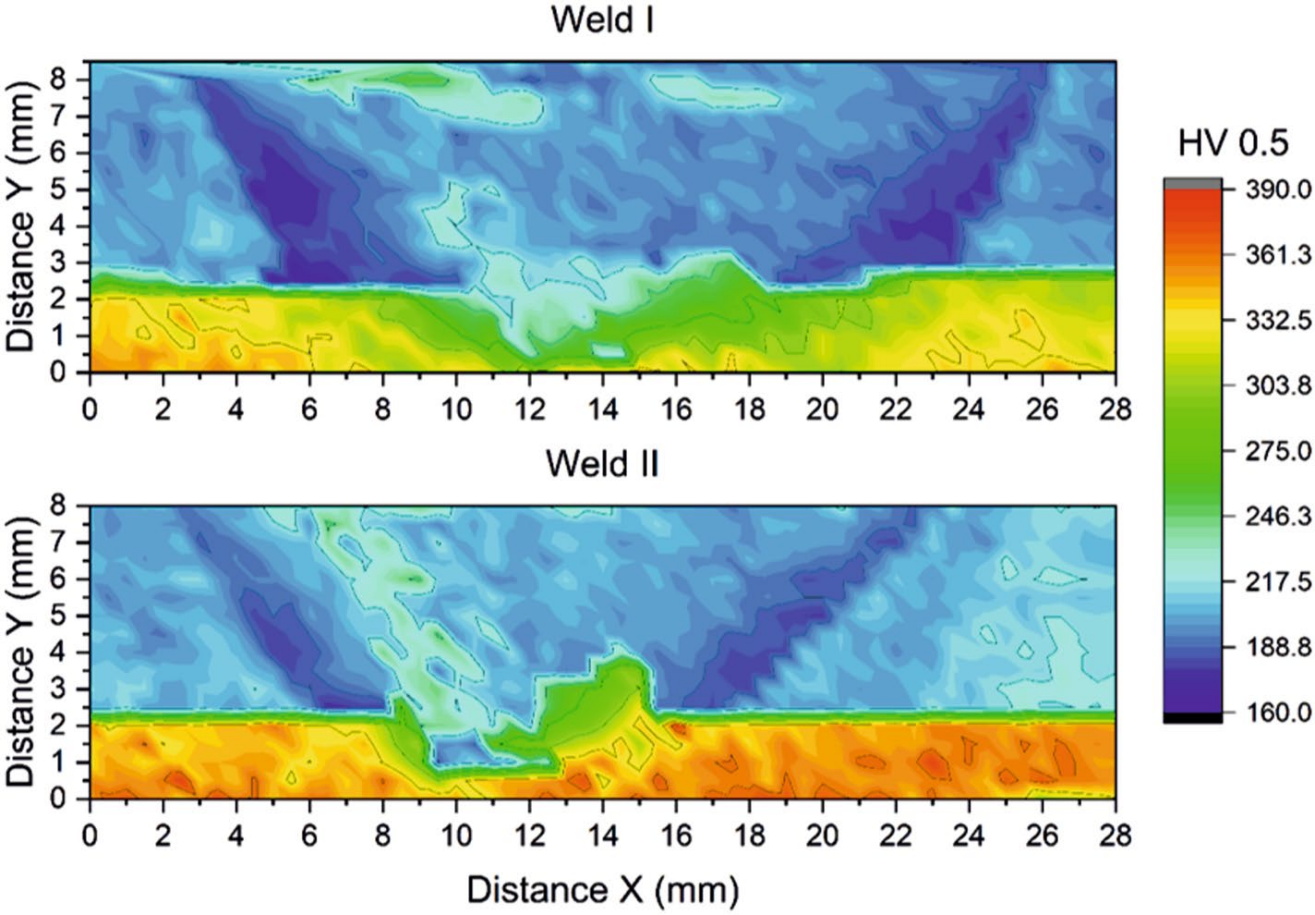
Microhardness maps of Weld I and Weld II, created via Origin, Version 2021. OriginLab Corporation, Northampton, MA, USA. Available: https://www.originlab.com/.

Vickers microhardness for Weld I and Weld II show differences in all zones of the Inconel 625 (Fig. 8). In the BM_625_ near the weld, values ranged between 310 and 360 HV for Weld I and Weld II, respectively. On the RS, where relatively coarse recrystallized Inconel 625 grains were found in SZ2_625_, the maximum values were 284 HV and 330 HV for Weld I and Weld II, respectively. The hardness values in the interface SZ are significantly lower due to the mixing between Inconel 625 and X65 steel, i.e., for Weld I, the values ranged from 218 to 303 HV, and for Weld II, even between 187 and 344 HV. The complex banded patterns, see Fig. 7a–d, contributed to the high hardness variations, as the BMs (X65 steel and Inconel 625) have different metallurgical and mechanical properties. On the steel side, the minimum hardness is found in the HAZ_X65_, varying between 172 and 180 HV, which agrees with results in the literature^14,24^. The hardness increases slightly with decreasing energy input while the width of the HAZ_X65_ decreases comparing the two welds. A small increase in microhardness was noted within the SZ_X65_, ranging between 200 and 250 HV for both welds. This slight improvement in microhardness in the SZ_X65_ is expected due to the severe deformation at elevated temperatures and high cooling rate, resulting in a more significant amount of bainite in the steel^21,33^. In the SZ _X65_, higher strain rates and associated plastic deformation caused higher hardness zones on the AS compared to the RS. The BM in the interface region showed a microhardness of 245 HV.

## Conclusions

The feasibility of orbital friction stir welding (FSW) for joining X65 carbon steel clad pipes with Inconel 625 was presented in this work. The results can be briefly summarized as follows:

I. The developed system was proven to produce FSW joints in one pass with full penetration and without volumetric defects. Friction-stir welded clad pipes of a total 9 mm wall thickness of API X65 steel and alloy 625 were produced at axial forces of 45–50 kN, tool rotational speeds of 400–500 rpm and a welding speed of 2 mm/s.
II. The orbital FSW process altered the microstructure of the base material. In API X65 PSL2 steel, three sub-regions were observed in the heat-affected zone with mainly ferrite microstructure. The predominant microstructures in the stir zone and hard zone, were bainite and acicular ferrite. In Inconel 625, two different regions were identified within the stir zone, one associated with refined recrystallized grains and higher hardness and other with grain growth and lower hardness. Weld II showed the same percentage of M/A as the base material, i.e., 0.2% and Weld I showed an increase of M/A to 0.5%.
III. Alternating bands of materials were found in the stir zone, following the tool flow pattern. A higher energy input seems to contribute to a homogeneous mixture of materials in the stir zone. In addition, higher energy input led to lower hardness and larger heat affected zones.
IV. The grain size of Inconel 625 varied along the stir zone. The concentration of high-angle grain boundaries increased from the base material (64.8%) to the stir zone (~ 93.0%), indicating that full recrystallization occurred in the Inconel 625. In the X65 steel, there was also a variation in the grain size, with a more remarkable grain growth in the SZ_x65_ compared to the X65 steel in the mixed interface. The high amount of grain boundaries with misorientation lower than 15° in the stir zone (~ 51%) and fewer peaks above 45° (~ 32%) suggest an increase in the amount of the bainite microstructure after FSW.

## Data availability

The datasets used and/or analyzed during the current study available from the corresponding author on reasonable request.
