# Author Correction: Feasibility of orbital friction stir welding on clad pipes of API X65 steel and Inconel 625

**DOI:** 10.1038/s41598-023-39136-z

**Published:** 2023-07-24

**Authors:** C. V. Amavisca, L. Bergmann, C. R. de L. Lessa, J. G. Schroeder, F. D. Ramos, G. V. B. Lemos, A. Reguly, B. Klusemann

**Affiliations:** 1 Institute for Production Technology and Systems, Leuphana University Lüneburg, Leuphana University Lüneburg, Universitätsallee 1, 21335 Lüneburg, Germany; 2 Federal University of Rio Grande do Sul (UFRGS), Av. Paulo Gama, 110, Porto Alegre, 90040-060 Brazil; 3 Institute of Materials Mechanics, Solid State Materials Processing, Helmholtz-Zentrum Hereon, Max-Plank -Straße 1, 21502 Geesthacht, Germany; 4 Federal Institute of Rio Grande do Sul (IFRS), R. Avelino Antônio de Souza 1730, Caxias do Sul, 95043-700 Brazil; 5 Federal University of Santa Maria (UFSM), Rod. Taufik Germano, Cachoeira do Sul, 301396503-205 Brazil

Correction to: *Scientific Reports,* 10.1038/s41598-023-37913-4, published online 1 July 2023

The Funding section in the original version of this Article was incomplete. It now reads:

“Open Access funding enabled and organized by Projekt DEAL. This publication was funded by the German Research Foundation (DFG).”

The original Article has been corrected.

